# Fast and Low-Overhead Time Synchronization for Industrial Wireless Sensor Networks with Mesh-Star Architecture

**DOI:** 10.3390/s23083792

**Published:** 2023-04-07

**Authors:** Zhaowei Wang, Tailiang Yong, Xiangjin Song

**Affiliations:** School of Electrical and Information Engineering, Jiangsu University, Zhenjiang 212013, China; wangzhaowei@ujs.edu.cn (Z.W.); songxiangjin@ujs.edu.cn (X.S.)

**Keywords:** industrial wireless sensor networks, time synchronization, mesh–star architecture, convergence, low-overhead

## Abstract

Low-overhead, robust, and fast-convergent time synchronization is important for resource-constrained large-scale industrial wireless sensor networks (IWSNs). The consensus-based time synchronization method with strong robustness has been paid more attention in wireless sensor networks. However, high communication overhead and slow convergence speed are inherent drawbacks for consensus time synchronization due to inefficient frequent iterations. In this paper, a novel time synchronization algorithm for IWSNs with a mesh–star architecture is proposed, namely, fast and low-overhead time synchronization (FLTS). The proposed FLTS divides the synchronization phase into two layers: mesh layer and star layer. A few resourceful routing nodes in the upper mesh layer undertake the low-efficiency average iteration, and the massive low-power sensing nodes in the star layer synchronize with the mesh layer in a passive monitoring manner. Therefore, a faster convergence and lower communication overhead time synchronization is achieved. The theoretical analysis and simulation results demonstrate the efficiency of the proposed algorithm in comparison with the state-of-the-art algorithms, i.e., ATS, GTSP, and CCTS.

## 1. Introduction

Industrial wireless sensor networks (IWSNs) are a special type of wireless sensor networks (WSNs) [1,2] that monitor the manufacturing process in a distributed manner, which can effectively reduce the deployment cost of traditional industrial Ethernet, Profinet, or other wired networks [3]. With the growing demand for large-scale ubiquitous production process monitoring in industrial networks, IWSNs have been extensively studied and applied. As the important supporting technology of fundamental service in IWSNs (e.g., low-power dormancy mechanism [4], multi-source data fusion [5], and time-slot-based transmission scheduling [6]) and new industrial applications (e.g., equipment fault tracing [7] and multi-robot cooperation [3]), time synchronization aims at keeping the IWSNs nodes be consistent in time. At the same time, plug and play in industrial network equipment is the general requirement of customizable flexibility manufacture in Industrie 4.0, that is, the faster the failure detection and recovery of network nodes, the better. In addition, IWSNs are mostly composed of resource-constrained nodes. Hence, convergence speed, robustness, and low overhead cannot be ignored for time synchronization in IWSNs.

In view of the significant role of time synchronization, many time synchronization algorithms in WSNs have been proposed for decades, which can be simply summarized as centralized and distributed time synchronization algorithms.

In centralized synchronization algorithms, such as *Flooding Time Synchronization Protocol* (FTSP) [8], *Per-Hop Delay Compensation scheme* (PHDC) [9], and *Rapid-flooding Multiple one-way broadcast Time-Synchronization* (RMTS) [1], reference nodes or root nodes are selected or designated as the time source for the entire network. This kind of algorithm usually maintains a spanning tree to realize a fast convergence rate, whereas, new nodes joining and node failure in the network may bring about frequent topology reconstruction. Therefore, poor robustness and scalability are inherent defects of centralized algorithms. To enhance the robustness and scalability, distributed synchronization algorithms, such as *Average TimeSync* (ATS) [2], *Maximum Time Synchronization* (MTS) [4], and *Gradient Time Synchronization Protocol* (GTSP) [10], achieve the entire network relative synchronization by adjusting the node’s local clock with neighbor’s information. Distributed algorithms are independent of topology and suitable for flexible network topologies and large-scale IWSNs. However, these algorithms require periodic iterations, which results in high cost, slow convergence, and low efficiency. In addition, variable and uncertain link delay may induce the synchronization error in distributed algorithms divergence.

As the algebraic connectivity of the network is positively related to the convergence rate of consensus-based time synchronization algorithms, *Virtual Topology-based Time Synchronization Protocol* (VTSP) [5] and *Multi-hop Average Consensus Time Synchronization* (MACTS) [11] increase the algebraic connectivity by forming virtual connections between nonadjacent nodes to accelerate algorithm convergence. Nevertheless, the information complexity and data redundancy inevitably raise. Clustering algorithms used in WSNs are effective methods of management, data integration, and information optimization. In [12,13], the synchronization process includes two parts: intra-cluster and inter-cluster with the overlapping clustered network topology. The cluster head bears more computing and communication costs, which reduces the communication traffic between nodes and accelerates the convergence rate of network synchronization. In spite of this, the inter-cluster synchronization accuracy is interfered by the intra-cluster synchronization accuracy, since intra-cluster synchronization is prior to inter-cluster synchronization. In addition, due to the lack of overlapping nodes, these algorithms tend to cause communication interruptions between adjacent clusters, which ultimately interferes with the time synchronization behavior between clusters.

Wireless networks for industrial automation-process automation (WIA-PA) is one of the representative industrial wireless standards. Different from the traditional distributed and clustered network topologies, WIA-PA supports the mesh–star hybrid architecture [14]. The upper mesh layer is formed by routing devices and has a strong anti-interference ability. The lower star layer can quickly broadcast the upper layer information to the local network with only one hop, while overcomes the uncertainty of the multi-hop transmission path. In this paper, we propose a fast and low-overhead time synchronization (FLTS) algorithm based on the mesh–star network structure. FLTS is initiated by routing nodes (including edge routing nodes that be responsible for accessing the star network) in the upper mesh layer, and the clock is adjusted in a distributed way. The nodes in the local star network only need to listen to the synchronization messages broadcasted by the edge routing nodes. The main contributions are as follows:

(1) We present a novel FLTS algorithm aimed at accelerating the convergence rate of time synchronization for IWSNs with mesh–star hybrid architecture that is widely used in WIA-PA industrial network. A two-layered time synchronization model is developed to improve the robustness and reduce the communication of synchronization.

(2) Normally, communication delay adversely affects the synchronization process. We propose a linear regression based relative clock skew estimation during the star layer time synchronization to make FLTS more resilient to communication delay. Furthermore, we prove the convergence and low communication overhead of FLTS theoretically.

The remainder of this paper is organized as follows. Section 2 describes the literature related to this work. The network model, clock model, and problem formulation are discussed in Section 3, while the proposed FLTS for IWSNs with mesh–star architecture is detailed in Section 4, including mesh layer synchronization, star layer synchronization, convergence analysis, and communication traffic. Section 5 carries out simulation to analyze and verify the performance of the proposed FLTS algorithm in terms of convergence rate and communication traffic under static and dynamic network, respectively. In Section 6, we conclude our work and discuss the future work.

## 2. Related Work

Consensus-based time synchronization algorithms have been widely studied for various demands in WSNs, e.g., robustness and scalability. Sommer et al. [10] proposed GTSP to accurately synchronize the clocks between neighbors in a completely decentralized fashion. It updates the local node’s clock states after receiving all neighbors’ beacons during a predefined synchronization period. In [2], adopting pseudo-periodic broadcast manner, each node in ATS broadcasts its time information based on its own clock and estimated the clock compensation values after receiving messages from any neighbor. He et al. [4] described a novel maximum-value-based consensus time synchronization algorithm, MTS. This algorithm can synchronize the skew and offset simultaneously with a faster convergence speed than ATS. In [15], a sequential least squares algorithm based on the recursive solution is applied to estimate the relative skew under bounded communications. The estimator calculates the initial value from direct solution with the same convergence performance. Subsequently, an average consensus-based scheme [2] utilizes the estimated skew to synchronize the network. Although the proposed algorithms [2,4,10,15] can meet different application requirements, time-consuming and message overload caused by iteration were not well handled.

In order to overcome the major drawback of consensus time synchronization algorithms, i.e., slow convergence caused by frequent iteration in large scale or sparse WSNs, various methods have been proposed. For instance, Xie et al. [16] formulated the synchronization in WSNs as a finite average consensus problem. A spanning tree, i.e., acyclic graph, is first constructed by a fast distributed depth-first-search algorithm. The synchronized clock rate and offset is the geometric mean and arithmetic mean, respectively. The convergence time is independent of the network topology and fully depends on the network diameter. However, generating a spanning tree needs additional computing and communication costs. The network topology varying with factory environment are also not conducive to the maintenance of a stable spanning tree.

Further, increasing the network connections, i.e., enhancing the algebraic connectivity, is an effective way to accelerate the convergence speed of consensus algorithms [17]. Phan et al. [5] designed VTSP based on GTSP, and attempted to improve the algebraic connectivity of network topology by creating virtual links between each node and its two-hop neighbors. But it needs additional optimization techniques to reduce the data redundancy and exclude edge nodes. Shi et al. [11] used the multi-hop communication model to generate virtual connections between non-adjacent nodes to against the slow convergence in ATS. In addition, a multi-hop controller is developed to simplify the message complexity and decrease the by-hop error accumulation. Nonetheless, each node has to forward its neighbor’s timing information to form virtual links, resulting in high information complexity.

Incorporating clustering techniques into distributed consensus algorithms are also frequently used to reduce the communication traffic and improve the convergence rate in WSNs time synchronization. For example, Wu et al. [12] firstly divided the network into overlapping clusters by low-energy adaptive clustering hierarchy algorithm, and then realized the intra-cluster synchronization and inter-cluster synchronization successively in average consensus. Wang et al. [13] proposed cluster-based maximum consensus time synchronization method (CMTS). The overlapping nodes undertake the message exchange between adjacent clusters. Besides, a Revised-CMTS is proposed to against the bounded communication delays. The synchronized virtual clock has the maximum value of initial clock parameters instead of the average value in [12]. To further reduce the overhead in CMTS, Wang et al. [18] proposed threshold-based intra-cluster synchronization to reduce the message broadcast and presented one-way synchronization in intra-cluster. In practical different line-of-sight (LOS) communication conditions, Chalapathi et al. [19] studied two-way synchronization in intra-cluster. It presents a simple and lower computational complexity method to estimate the relative clock parameters. Jia et al. [20] proposed threshold-based K-means clustering algorithm to achieve packet-efficient time synchronization. It utilizes the varying rate of all clocks to form clusters as well as to detect malicious node. The security of time synchronization has been strengthened, while the synchronization phase is not detailed.

Despite cluster-based synchronization algorithms are more message efficient and faster convergent, several deficiencies still exist. Clustered network is divided into two layers: inter-cluster layer and intra-cluster layer. The synchronization path from intra-cluster to inter-cluster renders the algorithm unable to resist node failure and movement [12]. Two-way synchronization consumes more communication energy in comparison to one-way synchronization [19]. In contrast with average consistency [2], the maximum-based algorithms [4] are more likely to be affected by communication interference, as the essence of average consistency is a low-pass filter. In addition, WIA-PA standard has defined the typical industrial network scenario: mesh–star hybrid architecture. In order to solve the problem of slow convergence speed of distributed algorithms, and enhance the robustness of cluster-based synchronization algorithms, we propose a clustered topology without overlapping nodes based time synchronization algorithm called FLTS, which aims to provide a new idea for the design of time synchronization and compensate for the deficiencies in literature.

## 3. System Model

Two-layered IWSNs with mesh–star architecture involved in this paper consists of a mesh level composed of routing nodes, and a star level composed of low-power sensing nodes. The network topology can be described as a connected undirected graph G=(V,E), where V=R∪S represents the set of nodes in IWSNs, and *R*, *S* define the node sets of the upper mesh network and the lower star network, respectively. For edge routing node *l*, l∈R, $C_{l}$ denotes the set of sensing nodes in star network, $S=\cup C_{l}$. E⊆V×V denotes the set of edges between nodes, (x,y)∈E means that node *x* and node *y* are adjacent. $N_{i}=\left\{j|\left(i,j\right)\in E,i\neq j\right\}$ is the set of neighbors for node *i*.

**Figure 1:**
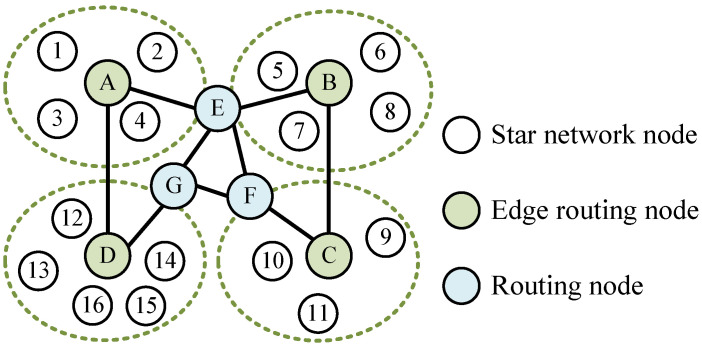
Schematic illustration of two-layered IWSNs. The number in figure denotes the node ID.

A typical IWNSs topology diagram is shown in Figure 1. Note that the nodes in the mesh level are divided into general routing nodes responsible for network interconnection (i.e., nodes *E*, *F*, and *G*) and edge routing nodes intended to access the star network (i.e., nodes *A*, *B*, *C*, and *D*). Frequent iterations are the key point that causes the slower convergence speed of distributed time synchronization algorithms. Consequently, the core idea of our design is to make fewer nodes bear the distributed iterations, in contrast, more nodes obtain synchronization by overhearing. For two-layered IWSNs with a mesh–star hybrid architecture, a few routing nodes with strong communication capability can be used to cover the industrial network with large-scale low-power sensing nodes. Furthermore, it employs the routing nodes participating in the synchronization iteration process to reduce the number of packet exchanges effectively.

In IWSNs, each node records its time by counting the periodic pulse signal generated by the crystal oscillator, which is called hardware time. While, even if two oscillators have the same nominal value, the beating frequency may vary due to the influence of manufacturing engineering, ambient temperature, crystal aging, and supply voltage [4]. As a consequence, the hardware time of the node would deviate over time. Here, node *i*’s hardware time $H_{i}\left(t\right)$ is described as a linear function of absolute time *t* [21]

(1) $$H_{i}\left(t\right)=\alpha_{i}t+\beta_{i}.$$

where, $\alpha_{i}$ is the hardware clock skew which represents the ratio between the actual frequency of the oscillator and its desired design at time *t*, $\beta_{i}$ is the initial hardware clock offset. Ideally, $\alpha_{i}$ and $\beta_{i}$ should be 1 and 0, respectively. Actually, crystal oscillators typically have a drift from the nominal value in the range of 10 ppm to 100 ppm [4], where $\alpha_{i}$ satisfies $1-p<\alpha_{i}<1+p$, p∈[10,100] ppm. Since the absolute time *t* is not available to the node, the values of $\alpha_{i}$ and $\beta_{i}$ cannot be obtained and adjusted directly [2,4]. Accordingly, we define the logical time to represent the synchronization time. The logical time $L_{i}\left(t\right)$ of node *i* is modeled as

(2) $$L_{i}\left(t\right)={\hat{\alpha }}_{i}H_{i}\left(t\right)+{\hat{\beta }}_{i}={\hat{\alpha }}_{i}\alpha_{i}t+{\hat{\alpha }}_{i}\beta_{i}+{\hat{\beta }}_{i}.$$

where, ${\hat{\alpha }}_{i}$ and ${\hat{\beta }}_{i}$ are the clock skew and offset compensations, respectively; ${\hat{\alpha }}_{i}\alpha_{i}$ and ${\hat{\alpha }}_{i}\beta_{i}+{\hat{\beta }}_{i}$ are logical clock skew and offset, respectively. The purpose of synchronization is to make the logical time of all nodes consistent by updating the clock skew and offset compensation parameters, namely,

(3) $$\left\{\begin{array}{c} {\hat{\alpha }}_{i}\alpha_{i}={\hat{\alpha }}_{j}\alpha_{j},\;\;\;\forall i,j\in V.\\ {\hat{\alpha }}_{i}\beta_{i}+{\hat{\beta }}_{i}={\hat{\alpha }}_{j}\beta_{j}+{\hat{\beta }}_{j},\;\;\;\forall i,j\in V. \end{array}\right.$$

To update the compensation values, it is necessary to understand the relationship between two hardware clocks, which means that acquiring the relative clock skew $\alpha_{ij}=\alpha_{j}/\alpha_{i}$. Since the hardware clock skew $\alpha_{i}$ cannot be acquired directly, the above formula is not suitable for the computation of relative clock skew $\alpha_{ij}$. To accomplish the objective of Equation (3), by collecting two consecutive timing messages containing hardware clock timestamps from adjacent nodes *j*, node *i* calculates the relative skew $\alpha_{ij}$ as follows

(4) $$\alpha_{ij}\left(t\right)=\frac{H_{j}\left(t\right)-H_{j}\left(t-1\right)}{H_{i}\left(t\right)-H_{i}\left(t-1\right)}.$$

Traditional distributed time synchronization algorithms synchronize the logical clocks of nodes by employing the linear consistency model [2,12,22]

(5) $$x_{i}\left(k+1\right)=x_{i}\left(k\right)+\sum_{j\in N_{i}}^{}\alpha_{ij}\left[x_{j}\left(k\right)-x_{i}\left(k\right)\right].$$

where $x_{i}\left(k\right)$ denotes the state of node *i*. Each node can use the information of its neighbor nodes to continuously update its state at each iteration. However, in large-scale networks, distributed synchronization requires frequent iterations to make the logical time of the whole network achieve an average value, which induces slow convergence speed and large communication traffic unfortunately.

## 4. Proposed FLTS Algorithm

To achieve fast convergent and low-overhead time synchronization for large-scale IWSNs, a novel FLTS algorithm, which is based on a two-layer mesh and star structure, is proposed in this paper. The FLTS algorithm consists of two parts: mesh layer time synchronization and star layer time synchronization, respectively. Therein, the routing nodes of the mesh network adopt the distributed consensus synchronization process, and the edge routing nodes send their updated time information to the connected star network nodes in realizing the synchronization of the whole network. In this section, the two steps of the proposed FLTS scheme are described in detail, and then the performance of the FLTS scheme is analyzed.

### 4.1. Mesh Layer Time Synchronization

Different from the MTS algorithm [13] with a fast convergence speed, due to the existence of communication delay, the maximum logical skew error increase continually, namely, the synchronization error diverges gradually, which eventually affects the synchronization of star network nodes in the next step. In order to have a certain anti-disturbance ability, we use ATS [2] as the basic time synchronization algorithm in the mesh layer (*lines 1–7 in Algorithm 1*). Specifically, the routing node *B* sends time information $<H_{B}\left(t\right),{\hat{\alpha }}_{B}\left(t\right),{\hat{\beta }}_{B}\left(t\right)>$ to their neighbor routing nodes during each predefined period *T*, and the neighbor routing node updates its logical skew and offset compensation parameters once receiving the message twice. It should be noted that the star network nodes do not participate in this step. Obviously, the algorithm can synchronize all routing nodes within a limited time.
**Algorithm 1** FLTS Algorithm**Input:** G=(V,E); ${\hat{\alpha }}_{i}\left(0\right)=1$, ${\hat{\beta }}_{i}\left(0\right)=0$, $\alpha_{ij}\left(0\right)=1$, i,j∈V; *T*; η, ρ, *v* **Output:** ${\hat{\alpha }}_{i}\left(t\right)$, ${\hat{\beta }}_{i}\left(t\right)$, i∈V1:**Upon routing node *****B*** **triggering a broadcast task**2:It broadcasts time information $<H_{B}\left(t\right)$, ${\hat{\alpha }}_{B}\left(t\right)$, ${\hat{\beta }}_{B}\left(t\right)>$ to neighbor routing nodes.3:**Upon routing node** ***A*** **receiving** ***B*****’s information**4:It stores $<H_{A}\left(t\right),H_{B}\left(t\right)>$, and calculates:$\alpha_{AB}\left(t^{-}\right)\leftarrow <H_{A}\left(t\right),H_{B}\left(t\right),H_{A}\left(t-1\right),H_{B}\left(t-1\right)>$5:Updates: $\alpha_{AB}\left(t\right)\leftarrow <\alpha_{AB}\left(t-1\right),\alpha_{AB}\left(t^{-}\right)>$6:Updates: ${\hat{\alpha }}_{A}\left(t\right)\leftarrow <{\hat{a}}_{A}\left(t-1\right),{\hat{\alpha }}_{B}\left(t\right),\alpha_{AB}\left(t\right)>$7:Updates: ${\hat{\beta }}_{A}\left(t\right)\leftarrow <{\hat{\beta }}_{A}\left(t-1\right),L_{A}\left(t\right),L_{B}\left(t\right)>$8:**if** *A* is the edge routing node, it **then**9: broadcasts time information $<H_{A}\left(t\right)$, ${\hat{\alpha }}_{A}\left(t\right)$, ${\hat{\beta }}_{A}\left(t\right)>$ to the star network.10:**end if**11:**Upon star network node** ***i*** **receiving** ***A*****’s information**12:It stores $<H_{i}\left(t\right),H_{A}\left(t\right)>$, and calculates:$\alpha_{iA}\left(t\right)\leftarrow <H_{A}\left(t\right),H_{i}\left(t\right),H_{A}\left(t-1\right),H_{i}\left(t-1\right)>$13:Updates: ${\hat{\alpha }}_{i}\left(t\right)\leftarrow <{\hat{\alpha }}_{A}\left(t\right),\alpha_{iA}\left(t\right)>$14:Updates: ${\hat{\beta }}_{i}\left(t\right)\leftarrow <{\hat{\beta }}_{i}\left(t-1\right),L_{A}\left(t\right),L_{i}\left(t\right)>$

#### 4.1.1. Skew Compensation in the Mesh Layer

When routing node *A* received two consecutive synchronization messages from its neighbor routing node *B*, including hardware time $H_{B}\left(t\right)$, skew compensation parameter ${\hat{\alpha }}_{B}\left(t\right)$, and offset compensation parameter ${\hat{\beta }}_{B}\left(t\right)$. Node *A* calculates the relative clock skew $\alpha_{AB}\left(t^{-}\right)$ with recorded local time synchronization:

(6) $$\alpha_{AB}\left(t^{-}\right)=\frac{H_{B}\left(t\right)-H_{B}\left(t-1\right)}{H_{A}\left(t\right)-H_{A}\left(t-1\right)}$$

Node *A* utilizes the newly calculated estimation and reserved prior value to update the existing relative clock skew:

(7) $$\alpha_{AB}\left(t\right)=\eta \alpha_{AB}\left(t-1\right)+\left(1-\eta \right)\alpha_{AB}\left(t^{-}\right)$$

where, η∈(0,1) is the artificially selected iteration weight parameter for the average operation. The initial relative clock skew $\alpha_{AB}\left(t\right)$ is 1. According to ${\hat{\alpha }}_{B}\left(t\right)$ and $\alpha_{AB}\left(t\right)$, node *A* updates ${\hat{\alpha }}_{A}\left(t\right)$

(8) $${\hat{\alpha }}_{A}\left(t\right)=\rho {\hat{\alpha }}_{A}\left(t-1\right)+\left(1-\rho \right){\hat{\alpha }}_{B}\left(t\right)\alpha_{AB}\left(t\right)$$

where, ${\hat{\alpha }}_{A}\left(t-1\right)$ is the logic skew compensation value of routing node *A* in the previous cycle and ρ∈(0,1) is the predefined iteration weight parameter.

#### 4.1.2. Offset Compensation in the Mesh Layer

After the convergence of skew compensation, all routing nodes have a consistent logical clock skew, and the logical clock offset of the routing nodes starts to converge gradually. The updating formula for offset compensation ${\hat{\beta }}_{A}\left(t\right)$ is

(9) $${\hat{\beta }}_{A}\left(t\right)={\hat{\beta }}_{A}\left(t-1\right)+\left(1-v\right)\left(L_{B}\left(t\right)-L_{A}\left(t\right)\right)$$

where, ${\hat{\beta }}_{A}\left(t-1\right)$ is the logic offset compensation value of routing node *A* in the previous cycle, $L_{A}$ is the logic time of routing node *A*, and v∈(0,1) is the weight parameter.

During the synchronization phase, each routing node broadcasts its own timing information periodically. Large amounts of data are frequently transferred between adjacent nodes, which inevitably leads to data conflicts. To tackle this, we can adopt time-division multiple access (TDMA) communication protocol and allocate transmission time slots for different messages according to time. Moreover, the asynchronous broadcast (also called pseudo-periodic) can be another effective way to avoid the conflict [2]. For each routing node, the real message broadcast instant is $t_{i}$. Since each node’s clock skew *i* is different, the real broadcast instant is asynchronous. Consequently, integrating TDMA communication and asynchronous broadcast can avoid data conflicts successfully.

### 4.2. Star Layer Time Synchronization

Star layer network synchronization adopts a one-way mechanism (*lines 8–14 in Algorithm 1).* The edge routing node broadcasts its time information to the local star network once updating the clock information, and node *i* in the star network records the time information when receiving the message. Node *i* calculates the relative clock skew $\alpha_{iA}\left(t\right)$ according to the received two consecutive time information, and then updates its skew and offset compensation ${\hat{\alpha }}_{i}\left(t\right)$ and ${\hat{\beta }}_{i}\left(t\right)$ as follows

(10) $${\hat{\alpha }}_{i}\left(t\right)={\hat{\alpha }}_{A}\left(t\right)\alpha_{iA}\left(t\right)$$

(11) $${\hat{\beta }}_{i}\left(t\right)={\hat{\beta }}_{i}\left(t-1\right)+\left(L_{A}\left(t\right)-L_{i}\left(t\right)\right)$$

Compared with existing time synchronization algorithms, FLTS algorithm constructs the mesh network by connecting routing nodes directly, which solves the problem of time synchronization failure caused by the loss of overlap nodes between neighboring clusters, due to energy consumption in the synchronization algorithms based on cluster network topology. The lower layer star network nodes only monitor the time information to synchronize passively, so the failure and movement of star network nodes would not interrupt the synchronization process, which ensures the robustness of FLTS.

The packets are subject to various interferences during transmission between nodes, namely, the communication delay will cause the packet receiving time distortion. As a consequence, the calculation of the relative skew as Equation (4) may mistaken, which further affects the updating of the logical clock in star network as Equations (10) and (11). As shown in Equation (12), the hardware time between two nodes obeys a linear relationship. To expand the practicability of FLTS, we adopt a linear regression method to estimate the relative clock skew against the communication delay.

(12) $$H_{A}\left(t\right)=\alpha_{A}\frac{H_{i}\left(t\right)-\beta_{i}}{\alpha_{i}}+\beta_{A}=\alpha_{iA}H_{i}\left(t\right)+\beta_{A}-\alpha_{iA}\beta_{i}$$

(13) $${\hat{\alpha }}_{iA}=\frac{\sum_{j=1}^{n}\left(H_{i}^{j}-{\bar{H}}_{i}\right)\left(H_{A}^{j}-{\bar{H}}_{A}\right)}{\sum_{j=1}^{n}{\left(H_{i}^{j}-{\bar{H}}_{i}\right)}^{2}}$$

where *n* is the number of samples required for linear regression, $\bar{H}$ is the mean value.

### 4.3. Convergence Analysis

The iterative process of FLTS is mapped into the state transition process of Markov chain [23].

(14) $$\begin{array}{cc} G & =\left(V,E\right)\to M_{G}\left(S,P\right)\\ V & =S,v_{1}=s_{1},\ldots ,v_{n}=s_{n}\\ W & =P,\omega_{ij}=p_{ij} \end{array}$$

where, the node $v_{i}$ in the network corresponds to each state $s_{i}$ of the Markov chain, the transition probability matrix of the Markov chain is represented by P, where $\omega_{ij}$ is obtained by:

(15) ωij=0(i,j)∉E,and vi≠vj1di+1(i,j)∈E,and vi=vj

where, $d_{i}$ is the degree of node *i*. The eigenvalues of the transition probability matrix P of $M_{G}$ satisfy $1=\lambda_{1}>\lambda_{2},\cdots ,\lambda_{n}>-1$. The convergence rate of $M_{G}$ depends on the second largest eigenvalue $\lambda_{2}$ of the matrix P, and its iteration number is $m=\frac{1}{log_{k}\left(\frac{1}{\lambda_{2}}\right)}$. The second largest eigenvalue $\lambda_{2}$ satisfies Cheeger’s inequality, namely $1-2\Phi \leq \lambda_{2}\leq 1-\frac{\Phi^{2}}{2}$. Where Φ is called the conduction coefficient of the Markov chain, which is approximately equal to $\frac{d}{2n}$, and then $1-\frac{d}{n}\leq \lambda_{2}\leq 1-\frac{d^{2}}{8n^{2}}$. *d* represents the average number of node’s neighbors, and *n* is the number of nodes in the network. In a large-scale network, $\frac{d}{n}$ and $\frac{d^{2}}{8n^{2}}$ approach 0, the convergence speed is $m\approx \frac{1}{1-\lambda_{2}}$, and $\frac{n}{d}\leq m\leq \frac{8n^{2}}{d^{2}}$.

For the synchronization process in the mesh layer, there are *N* routing nodes, and each routing node has *M* neighbor routing nodes. Although the network is not a recurrent network, the whole network is deployed randomly and can be regarded as a uniform-like network. Hence, the conclusion of the iteration number *m* obtained above is also applicable to a uniform-like network, namely, $\frac{N}{M}\leq m\leq \frac{8N^{2}}{M^{2}}$. Furthermore, star network nodes only need to synchronize with the edge routing nodes in one-way, and then a finite time synchronous convergence is achieved.

### 4.4. Communication Traffic

During the synchronization in the star network, each edge routing node sends time information to the star network nodes after updating its logical clock. During each synchronization period, the edge routing node *i* and its neighbors announce their local time information once. As a result, the packet exchange times in each iteration of the mesh layer and the star layer are *N* and $\sum_{i=1}^{L}D_{i}$, respectively, where $D_{i}$ is the number of routing neighbors for edge routing node *i*, *L* is the number of edge routing nodes. Therefore, FLTS algorithm needs $N+\sum_{i=1}^{L}D_{i}$ packet exchanges for network-wide synchronization. Concerning to the analysis in distributed consensus time synchronization algorithms, the comparison of FLTS, GTSP, and CCTS on communication traffic in a single-step iteration is shown in Table 1.

It should be noted that ${\tilde{D}}_{i}$ represents the number of neighbor clusters in CCTS, $d_{i}$ is the ordinary neighbors’ number in GTSP, and *n* is the number of nodes in the network. Obviously, L≤N≪n and $D_{i}≃{\tilde{D}}_{i}$, FLTS is communication efficient. Through the above analysis, increasing the neighbors of the routing node, namely, improving the network density, or reducing the routing nodes, can effectively reduce the number of network iterations.

## 5. Simulation Results

In this section, a Matlab simulation is performed to verify the effectiveness of the FLTS algorithm compared with ATS, GTSP, and CCTS. The initial clock compensation parameters are set as ${\hat{\alpha }}_{i}=1,{\hat{\beta }}_{i}=0$, $\alpha_{ij}=1$. Assume that the hardware clock skew and offset $\alpha_{i}$ and $\beta_{i}$ of each node are randomly selected from the set [0.9999,1.0001] and [0,0.0002] [4], respectively; T=1 s, η=0.2, ρ=0.5, and v=0.5 [2].

### 5.1. Static Network

**Figure 2:**
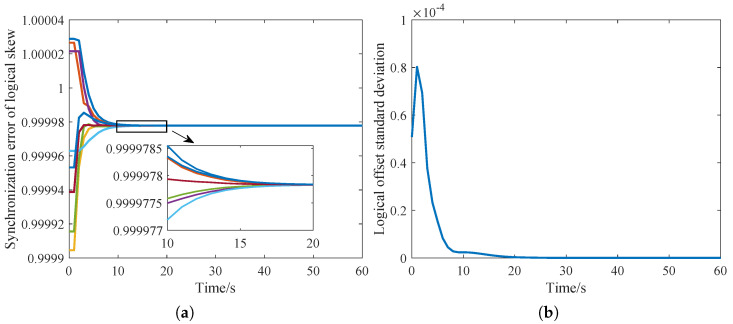
The convergence of the logical clock in the mesh layer. (**a**) Logical clock skew; (**b**) Logical clock offset.

In this section, we consider a static network with delay-free case. A typical two-layered IWSNs is built, where the mesh layer is composed of 8 edge routing nodes randomly distributed, and each edge routing node connects 3 star network nodes. In Figure 2a, the logical clock skew of the mesh network gradually converges after about 10 s. After about 20 rounds, the logical skew of the routing nodes all converge to the same value. Figure 2b shows that in the initial stage, the logic skew has not converged, resulting in the rise of the logic offset synchronization error. After the logical skew is synchronized, all routing nodes converge to the same logical clock.

**Figure 3:**
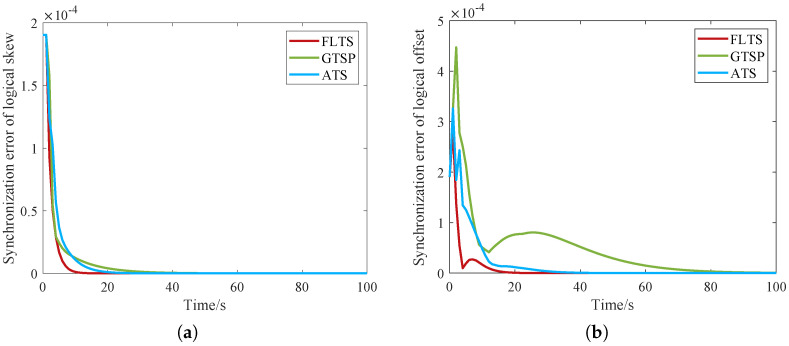
Convergence comparison of the logical clock in whole network. (**a**) Logical clock skew; (**b**) Logical clock offset.

Figure 3a shows that the logical clock skew convergence speed of FLTS is faster than that of the traditional distributed algorithm. As the convergence of the logic clock offset depends on the logic clock skew, the faster the logic clock skew converges, the faster the logic clock offset of the entire network converges, as shown in Figure 3b. Obviously, FLTS converged after about 20 s, while ATS and GTSP are still synchronizing. Moreover, the logical offset synchronization error is closely related with the product of skew synchronization error and time *t*. Although the skew synchronization error is monotonically decreasing, time *t* is monotonically increasing. The curves in Figure 3b would fluctuate during the convergence process.

**Figure 4:**
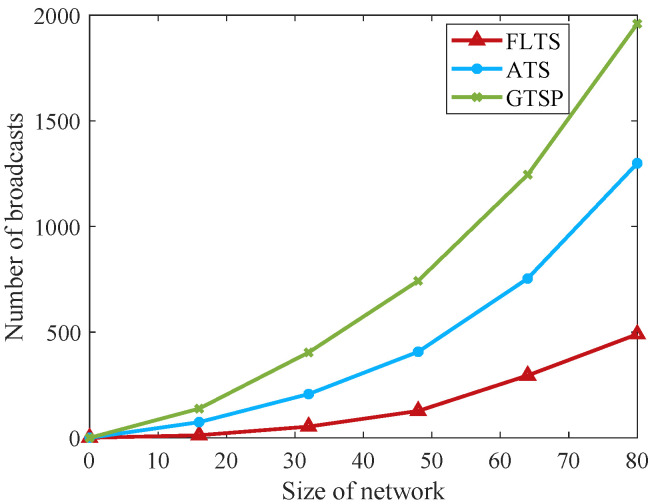
Comparison of communication overhead.

In ATS and GTSP, the communication range of nodes determines the number of their neighbors, which in turn affects the convergence speed of the network. Furthermore, it requires that each node needs to have a certain communication capability while increasing the energy consumption of the network dramatically. In contrast, FLTS just needs to ensure the communication range of the routing node, and the routing node itself is more powerful than ordinary nodes. Additionally, ATS is an asynchronous synchronization algorithm, and the node triggers the iterations once receiving the broadcast information from the neighbors. As a result, the node updates the clock in a waiting manner to enter the broadcast cycle, which produces frequent iteration, that is, frequent packet interaction. GTSP is a synchronous synchronization algorithm, which updates clock parameters after receiving the time information from all neighboring nodes during the synchronization period. This algorithm can provide accurate clock synchronization between neighbors, but with a slow convergence speed. In FLTS, the edge routing node can synchronize the star network nodes through twice broadcasts, which reduces the number of iterations greatly. As can be seen from Figure 4, with increasing the network scale, the number of packet exchanges of FLTS is significantly smaller than that of ATS and GTSP algorithms. In the simulation environment, the number of nodes in the network is set to 16, 32, 48, 64, and 80 (there are 4, 8, 12, 16, and 20 routing nodes in FLTS).

**Figure 5:**
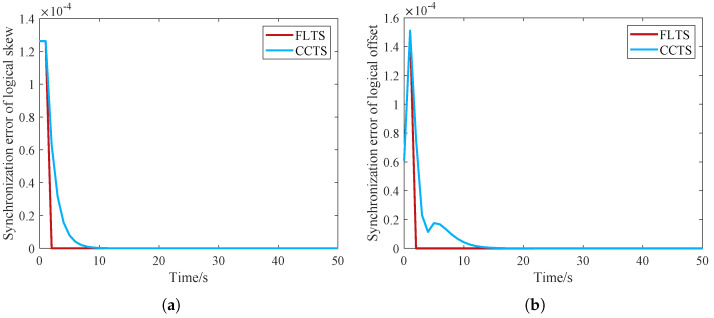
Convergence comparison of the logical clock in intra-cluster synchronization. (**a**) Logical clock skew; (**b**) Logical clock offset.

**Figure 6:**
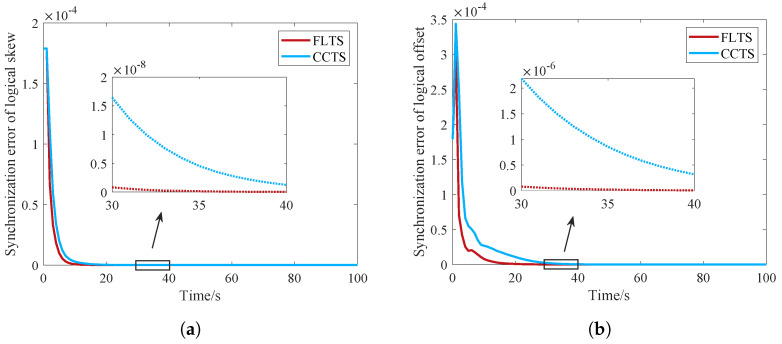
Convergence comparison of the logical clock in inter-cluster synchronization. (**a**) Logical clock skew; (**b**) Logical clock offset.

To further illustrate the superiority of FLTS, we compare the convergence performance of FLTS with CCTS in intra-cluster synchronization and inter-cluster synchronization, respectively. It should be noted that the inter-cluster synchronization here represents the synchronization of the whole network. In FLTS, the edge routing nodes which are responsible for star network access to mesh network, broadcast the clock compensation parameters to sensing nodes to realize synchronization in star networks. The nodes in star network only need to monitor and synchronize with the edge routing nodes with two consecutive packets. Instead, CCTS’s cluster members need to execute successive iterations to synchronize with the cluster head. Although FLTS and CCTS all work in average consensus based upper layer synchronization, the difference of lower synchronization renders them with performance difference. As shown in Figure 5 and Figure 6, FLTS show faster convergence speed than CCTS, which also means that the communication and computational overhead can be reduced effectively.

### 5.2. Dynamic Network

**Figure 7:**
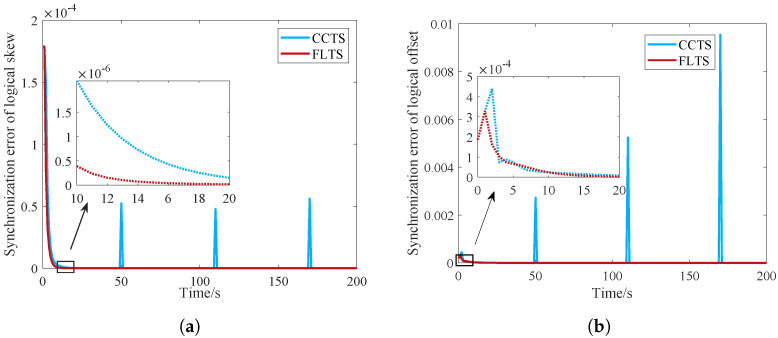
Convergence comparison of the logical clock against node mobility. (**a**) Logical clock skew; (**b**) Logical clock offset.

In the following simulations, we further evaluate the performance of FLTS with node mobility and random delay cases. During the implementation of CCTS, each node maintains two sets of clock compensation parameters, which are used to compensate the intra-cluster logical clock and the inter-cluster logical clock. Among them, inter-cluster synchronization depends on the realization of intra-cluster synchronization entirely. The fluctuation of intra-cluster synchronization would cause serious interference on the inter-cluster synchronization status. Node mobility is an non-negligible issue in industrial networks. When a node move from a cluster to another during the synchronization process of FLTS, its clock compensation parameters remains the same as the previous cluster, which may break the synchronization balance between the new cluster and the old cluster. In the simulation, the positions of the three randomly selected nodes move at the moments 50, 110 and 170 in succession. In Figure 7, in CCTS, the error of logical clock skew fluctuates at the moment of node movement, which seriously affects the synchronization of the network. In contrast, the star network nodes in FLTS listen the time information of the edge routing nodes directly, and do not care about their position changes, so they are not affected by the node movement.

**Figure 8:**
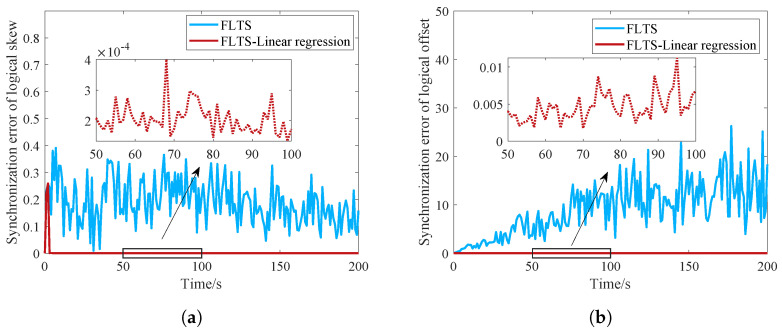
Convergence comparison of the logical clock against communication delay. (**a**) Logical clock skew; (**b**) Logical clock offset.

In the follows, we discuss the performance of FLTS under the impact of random communication delay. In FLTS, the mesh layer adjusts the relative clock skew by average iteration, which is essentially a low-pass filter. It can suppress part of the impact of delay on the calculation of the relative clock skew. However, if there is no measures taken during the synchronization at the mesh level, the synchronization divergence is inevitalbe. In the simulation, set the communication delay with mean $2.5\times 10^{-4}$ and variance $1\times 10^{-8}$. As shown in Figure 8, the linear regression method can effectively reduce the synchronization error of the logical clock skew and offset. Nevertheless, the network cannot achieve absolute synchronization under communication delay, the synchronization error of logical clock skew still affect the logical offset synchronization. The maximum synchronization error of the whole network gradually increases with time, however, the effectiveness of linear regression is still reflected.

## 6. Conclusions

A novel time synchronization algorithm, i.e., FLTS, is proposed to solve the problems of slow convergence and high cost in large-scale IWSNs. The main idea of FLTS is to perform the synchronization process into two layers, that is, the average iteration in the mesh layer and one-step monitoring in the star layer. It can reduce communication traffic and accelerate convergence effectively. Extensive analysis and simulations demonstrate the effectiveness of FTLS algorithm in comparison with ATS, GTSP, and CCTS.

Although FLTS is robust under ordinary node mobility in the star layer, the impact of the routing nodes’ statuses in the mesh layer cannot be ignored. For example, when an edge routing node moves or breaks down, the nodes in the star layer accessing to the edge routing node may lose synchronization with the network. It requires additional overhead to maintain the stability and reliability of the upper mesh network. Moreover, the communication delay in industrial network is a fundamental restriction in time synchronization. It is difficult to establish an accurate delay model in the complex factory environment. Various delay distribution models have been proposed, e.g., random bounded delay, exponential delay, normal distribution delay, et al. Meanwhile, most research results including FLTS, are verified through simulation. How effective the algorithm is in a real factory environment still needs further verification. Future directions include solving the influence of mesh layer network fluctuation, experimental validation, and extending the idea to more complicated time-delay industrial networks.

## Figures and Tables

**Table 1:** Communication Overhead Comparison.

Algorithms	Communication Overhead
GTSP [10]	$\sum_{i=1}^{n}\left(1+d_{i}\right)$
CCTS [12]	$n+\sum_{i=1}^{N}\left(1+3{\tilde{D}}_{i}\right)$
FLTS	$N+\sum_{i=1}^{L}D_{i}$

## Data Availability

Not applicable.
